# A Retrospective Study of the Characteristics and Clinical Significance of A-Waves in Amyotrophic Lateral Sclerosis

**DOI:** 10.3389/fneur.2017.00515

**Published:** 2017-09-28

**Authors:** Jia Fang, Liying Cui, Mingsheng Liu, Yuzhou Guan, Qingyun Ding, Dongchao Shen, Dawei Li, Hongfei Tai

**Affiliations:** ^1^Department of Neurology, The Second Xiangya Hospital, Central South University, Changsha, China; ^2^Department of Neurology, Peking Union Medical College Hospital, Chinese Academy of Medical Sciences, Peking Union Medical College, Beijing, China; ^3^Neuroscience Center, Chinese Academy of Medical Sciences, Beijing, China

**Keywords:** A-wave, amyotrophic lateral sclerosis, F-wave, disease progression rate, revised amyotrophic lateral sclerosis Functional Rating Scale, split-hand, split-leg

## Abstract

A-wave was observed in patients with motor neuron disease (1). However, data on the characteristics and clinical significance of A-waves in patients with amyotrophic lateral sclerosis (ALS) have been scarce. The F-wave studies of 83 patients with ALS and 63 normal participants which were conducted previously at the Department of Neurology in Peking Union Medical College Hospital were retrospectively reviewed to determine the occurrence of A-waves in ALS. A-waves occurred more frequently in ALS patients than in normal controls. For the median and peroneal nerves, the frequencies of nerves with A-waves and frequencies of patients with A-waves were comparable between the ALS patients and normal controls. For the ulnar and tibial nerves, the frequencies of nerves with A-waves and frequencies of patients with A-waves were significantly increased in the ALS patients compared with those of the normal participants. Disease progression rate was slower in the ALS patients with A-waves (0.73 ± 0.99) than that in the ALS patients without A-waves (0.87 ± 0.55, *P* = 0.007). No correlations were found between the amplitudes of F-waves with A-waves and those of A-waves in the ulnar nerves (*r* = 0.423, *P* = 0.149). No correlations were found between the persistence of F-waves with A-waves and the persistence of A-waves in the ulnar nerves as well (*r* = 0.219, *P* = 0.473). The occurrence of A-waves may indicate dysfunction of lower motor neurons and possibly imply a relatively slower degenerative process.

## Introduction

A-waves are compound muscle action potentials that follow the M-wave with a constant shape and latency, which are detected during F-wave studies (2). Recently, the term A-waves has been used in a more general manner for abnormal late responses that are not axon reflexes and are recorded in motor nerve conduction studies using supramaximal stimuli (2, 3). A-wave is a relatively common finding during routine F-wave studies using standard stimulation and recording techniques. It was proposed that A-waves were indicator of dysfunction of peripheral nerves, especially of generalized and demyelinating origin (3, 4). There are numerous studies focusing on the presence of A-waves in demyelinating neuropathies, especially in Guillain–Barré syndrome. It was reported that A-wave could be observed in patients with motor neuron disease (1), though demyelination is the crucial underlying pathophysiologic correlate of supramaximally stimulated A-waves (4). Data on the characteristics and clinical significance of A-waves in patients with amyotrophic lateral sclerosis (ALS) are scarce. The present study aimed to investigate the occurrence and characteristics of A-waves in ALS patients.

## Materials and Methods

The retrospective study reviewed the electrodiagnostic studies performed on patients with ALS and normal volunteers at Department of Neurology, Peking Union Medical College Hospital from September 2014 to August 2015. Neurophysiological tests from 83 ALS patients and 63 normal participants were retrieved for reanalysis. All the 83 patients with ALS, 52 males and 31 females with mean age 49.53 ± 9.69 years (range 28–79) were diagnosed as having definite, probable, or laboratory-supported probable ALS according to the revised El Escorial criteria (5). The ALS patients were evaluated clinically using the revised ALS Functional Rating Scale (ALSFRS-R) score (6). Disease progression rate was calculated as (48 − ALSFRS-R)/disease duration (months) (7). Forty-eight was the total score of ALSFRS-R. Disease duration was defined as time between symptom onset and date of evaluation. As normal control group, the occurrence of A-waves during F-wave study was investigated in 63 healthy subjects, 34 males, and 29 females (mean age 46.25 ± 11.46 years, range 27–73). For the ALS patients and healthy controls, participants with diabetes, uremia, chronic alcohol abuse, rheumatoid arthritis, or other disorders known to cause polyneuropathies had been excluded.

### Nerve Conduction Studies

The electrodiagnostic studies were performed on a Viking IV electromyography system (Nicolet Biomedical, Madison, WI, USA). Motor nerve conduction studies and F-waves were performed in the median, ulnar, peroneal, and tibial nerves bilaterally using surface electrodes with a standardized technique. The active electrode was placed over the middle of the muscle belly (abductor pollicis brevis, abductor digiti minimi, abductor halluces brevis, and extensor digitorum brevis, respectively) and the reference electrode was placed on the tendon distally. Stimulation was performed at the wrist (70 mm proximally from the recording electrode), elbow, and axilla for the median nerve; at the wrist (70 mm proximally from the recording electrode), below the elbow, above the elbow, and axilla for the ulnar nerve; at the ankle and popliteal fossa for the tibial nerve; and the ankle, below the fibular head, and above the fibular head for the peroneal nerve. Parameters including CMAP and CMAP amplitude ratio of the median, ulnar, tibial, and peroneal nerves were analyzed.

### F-Wave and A-Wave Study

The median, ulnar, tibial, and peroneal nerves were stimulated 70 mm proximally to the recording electrode using electrical stimuli of 0.1 ms duration at a frequency of 1 Hz. A 100 supramaximal stimuli were employed to elicit F-waves. The sweep speed was 10 ms/div for leg nerves and 5 ms/div for arm nerves. The minimum F-wave latencies, mean F-wave amplitude, and F-wave persistence were assessed.

F-wave recordings of 664 nerves in patients with ALS were analyzed with regard to the occurrence of A-waves. A-waves were defined according to the following criteria: (1) supramaximally elicited reproducible waveforms with amplitudes greater than 0.05 mV, clearly separated from the M-wave; (2) relatively constant latency, amplitude, and morphology of the response with every electrical stimuli; and (3) occurrence in at least 35% stimuli (1, 8). Responses with characteristics of an A-wave occurring within the F-response or instead of it were accepted as A-waves. The A-waves were grouped into the following groups according to their relationships to F-waves: (1) A-waves preceding the F-waves, (2) A-waves intermixing with F-waves, (3) A-waves following the F-waves, and (4) multiple A-wave (2). The electrophysiological features of multiple A-waves included: (1) greater than three phases; (2) desynchronization (i.e., temporal dispersion); or (3) two or more discreet biphasic potentials (1). The frequency of A-waves in patients with ALS, differences in clinical characteristics between patients with and without A-waves, the number of nerves showing A-waves in a single patient, the relationship of A-wave and F-wave, etc. were analyzed.

To find whether there was any correlation between characteristics of A-wave and F-wave in ALS. In the present study, we compared the amplitude and persistence of F-waves with A-waves with those of F-waves without A-waves in ulnar nerves, and tried to find whether there were any correlations between them. The amplitude and persistence of F-waves or A-waves in the ulnar nerves were measured. A peak-to-peak deflection from baseline of at least 40 µV was accepted as an F-wave. The amplitudes of F-waves and A-waves were measured from peak-to-peak. The persistence of F-waves and A-waves was defined as the number of F-waves and A-waves per 100 stimuli. To address the implications of different A-wave latencies, the CMAP amplitudes of nerves with early A-waves with those of nerves without early A-waves were compared in the median, ulnar, tibial, and peroneal nerves, respectively. The research protocol was approved by the ethics committee of Peking Union Medical College Hospital and adhered to the principles of Declaration of Helsinki. Informed consent was not required as this was a retrospective study.

### Statistics Analysis

The Kolmogorov–Smirnov test was used to test the normality of the data. The levene test was used to test the homogeneity of variance between different groups. If the data were normally distributed, a Student’s *t*-test was performed. If the data were not normally distributed, a Mann–Whitney *U* test was used. A χ^2^ test was used for categorical data. Findings were considered statistically different when *P* < 0.05. All the data were expressed as the mean ± SD. All the statistical tests performed were two-sided and were conducted using SPSS for windows, version 21.0 (SPSS, Inc., Chicago, IL, USA).

## Results

Table 1 showed the demographic features and motor nerve conduction studies of ALS patients and normal control participants. Though the frequency of A-waves increased with age, ages were comparable between normal control participants and ALS patients in the present study. Compared with the normal controls, the CMAP amplitudes of the APB and the ADM as well as the APB/ADM CMAP amplitude ratios of the ALS patients were significantly lower. The CMAP amplitudes of the AHB and the EDB as well as the EDB/AHB CMAP amplitude ratios of the ALS patients were significantly lower than those of the normal controls.

**Table 1:** Demographic features and motor nerve conduction study of amyotrophic lateral sclerosis (ALS) patients and normal controls.

	ALS	Normal control	*P*-values
Age	49.53 ± 9.69	46.25 ± 11.46	0.064
Height	167.13 ± 6.98	167.38 ± 7.60	0.838
Gender ratio (F:M)	30:53	28:35	0.310
APB CMAP	6.50 ± 4.96	14.24 ± 3.28	<0.001
ADM CMAP	8.88 ± 4.56	16.22 ± 3.05	<0.001
APB/ADM	0.76 ± 0.72	0.90 ± 2.44	<0.001
AHB CMAP	12.43 ± 6.45	14.84 ± 5.52	0.001
EDB CMAP	4.97 ± 2.95	7.40 ± 3.21	<0.001
EDB/AHB	0.56 ± 1.28	0.54 ± 0.31	0.001

*APB, abductor pollicis brevis; ADM, abductor digiti minimi; AHB, abductor halluces brevis; CMAP, compound muscle action potential; EDB, extensor digitorum brevis*.

**Figure 1:**
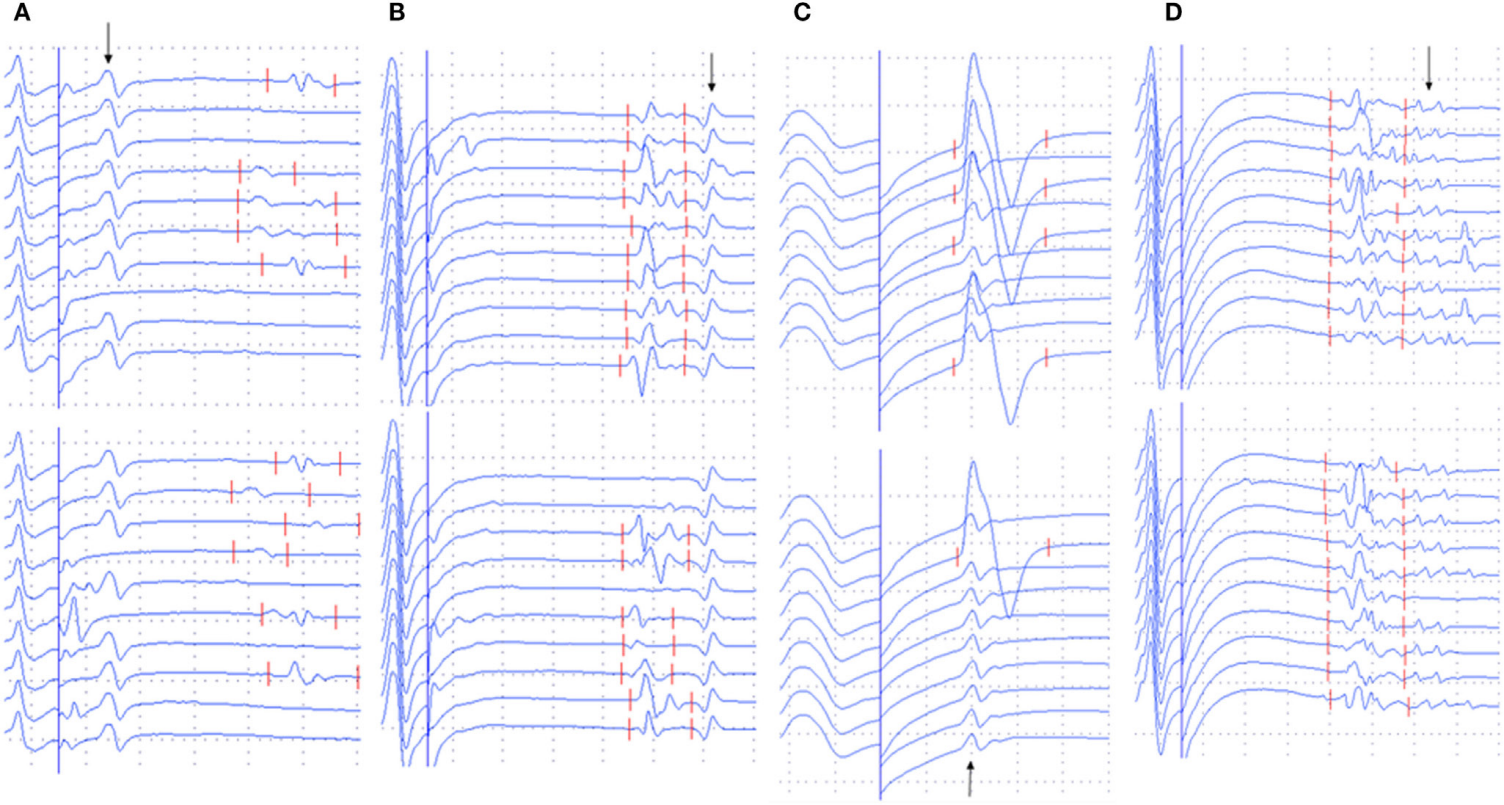
Examples of A-waves (early A-waves, late A-waves, A-waves mixed with F-waves, and multiple A-waves) recorded during F-wave studies in patients with amyotrophic lateral sclerosis (ALS). **(A)** Early A-waves occurring before the F-waves with right tibial nerve stimulation in a patient with ALS. **(B)** Late A-waves following the F-waves with stimulation of the left tibial nerve in a patient with ALS. **(C)** A-waves occurring among the F-waves with left median nerve stimulation in a patient with ALS. **(D)** Multiple A-waves recorded from the right tibial nerve in a patient with ALS. The A-waves are marked by an arrow. Dural gain is used; during the M-wave, the gain is 5 mV/div, and during the late responses, the gain is 0.5 mV/div.

The occurrence of different types of A-waves in ALS patients and normal controls was given in Table 2. In the normal control participants, A-waves were detected in 19 nerves of 17 subjects. A-waves were more often recorded in the lower extremity nerves (84%) and found less commonly in the upper extremity nerves (16%) in healthy subjects. In normal participants, the early A-waves were by far most common. While in the ALS patients, late A-waves were observed most commonly. Examples of different kinds of A-waves were shown in Figure 1. Of all patients with ALS, 49% had at least one nerve with A-waves, significantly increased compared with the normal controls (χ^2^ = 7.514, *P* = 0.006). The frequency of A-waves in the ALS patients in the present study was similar to a previous study (8).

**Table 2:** Occurrence of different types of A-waves in patients with amyotrophic lateral sclerosis (ALS) and normal controls.

	ALS	Normal control
Nerves with A-wave	61	19
Early A-wave	21/61 (36%)	13/19 (68%)
Late A-wave	26/61 (43%)	6/19 (32%)
A-wave intermixed with F-wave	7/61 (11%)	0 (0%)
Multiple A-wave	1/61 (2%)	1/19 (5%)
A-wave in the absence of F-wave	6/61 (10%)	0 (0%)

For the median and peroneal nerves, the frequency of nerves with A-waves and the frequency of patients with A-waves were comparable between the ALS patients and the normal controls. For the ulnar and tibial nerves, the frequencies of nerves with A-waves and the frequencies of patients with A-waves were significantly increased in the ALS patients compared with those of the normal participants. The frequencies of A-waves detected in one nerve in the ALS patients were similar to those in the normal controls (ALS 28/83, NC 16/63, χ^2^ = 1.183, *P* = 0.277), while the frequencies of A-waves recorded in more than one nerve in the ALS patients were significantly increased compared with those in the normal controls (ALS 13/83, NC 1/63, χ^2^ = 8.184, *P* = 0.004).

The characteristics of A-waves in the ALS patients and the normal controls were shown in Table 3. The comparisons of the clinical characteristics between the ALS patients with and without A-waves were shown in Table 4. Compared with the ALS patients without A-waves, the ALS patients with A-waves showed a slower disease progression rate. There were no significant differences between the amplitudes of F-waves with A-waves (363.08 ± 320.38 µV) and those of F-waves without A-waves (381.42 ± 291.55 µV, *P* = 0.897). No significant differences were found between the persistence of F-waves with A-waves (49.12 ± 47.65%) and those of F-waves without A-waves (70.55 ± 34.82%, *P* = 0.187). No correlation was found between the amplitudes of F-waves with A-waves and the amplitudes of A-waves in the ulnar nerves (*r* = 0.423, *P* = 0.149). No correlation was found between the persistence of F-waves with A-waves and the persistence of A-waves in the ulnar nerves as well (*r* = 0.219, *P* = 0.473). As demonstrated in Table 5, no differences were found in the CMAP amplitude between nerves with early A-waves and nerves without early A-waves in the median, ulnar, tibial, and peroneal nerves, respectively.

**Table 3:** Characteristics of A-waves in patients with amyotrophic lateral sclerosis (ALS) and normal controls.

	ALS	Normal control	*P*-values
**Frequency of nerves with A-waves**			
Median nerve	8/166	2/126	0.238
Ulnar nerve	11/166	1/126	0.013
Tibial nerve	35/166	13/126	0.014
Peroneal nerve	7/166	3/126	0.596
**Frequency of patients with A-waves**			
Median nerve	8/83	2/63	0.230
Ulnar nerve	11/83	1/63	0.011
Tibial nerve	28/83	12/63	0.049
Peroneal nerve	6/83	3/63	0.790

**Table 4:** Clinical features of A-wave positive vs. A-wave negative patients with amyotrophic lateral sclerosis.

Demographic	A-wave positive	A-wave negative	*P*-values
Number of patients	41	42	–
Men (women)	28/13	24/18	0.294
Disease duration (months)	17.95 ± 13.87	14.87 ± 8.37	0.314
ALSFRS-R	39.12 ± 5.77	37.08 ± 5.43	0.071
Disease progression rate	0.73 ± 0.99	0.87 ± 0.55	**0.007**

*Values are mean ± SD. Values with significant differences printed in bold characters*.

*ALSFRS-R, revised ALS Functional Rating Scale*.

**Table 5:** Comparisons in CMAP amplitudes between nerves with early A-waves and nerves without early A-waves.

	Nerve with early A-waves	Nerve without early A-waves	*P*-values
APB CMAP (μV)	2.85 ± 0.35	6.52 ± 4.96	0.327
ADM CMAP (μV)	11.50 ± 3.65	8.77 ± 4.61	0.270
AHB CMAP (μV)	10.52 ± 5.56	12.60 ± 6.52	0.268
EDB CMAP (μV)	8.43 ± 6.13	4.90 ± 2.85	0.276

*APB, abductor pollicis brevis; ADM, abductor digiti minimi; AHB, abductor halluces brevis; CMAP, compound muscle action potential; EDB, extensor digitorum brevis*.

## Discussion

A-waves are compound muscle action potentials that follow the M-waves with a constant shape and latency (2). According to the present study, A-waves could be seen as additional signs of pathology in patients with ALS. The significantly increased number of A-waves in ALS patients compared with that in the normal controls implied that the presence of A-waves did suggest a pathological process. There are several possible pathophysiologic mechanisms responsible for the generation of A-waves, including proximal reexcitation of the axon, myo-axonal ephapses, or transaxonal ephaptic transmission (9, 10). The precise mechanisms of supramaximally elicited A-waves remain to be determined. It was assumed that the site of a nerve lesion was not the site of origin of the A-wave. It was reported that in ALS, there was secondary demyelination in the proximal nerve segments or nerve roots and proximal axonal swelling (11). The occurrence of A-waves may be also related to neuropathic changes of alpha motor neurons (2).

In ALS patients, there was differential involvement between different muscles (12, 13). Lower motor neuron degeneration in abductor pollicis brevis was faster than that in abductor digiti minimi, while motoneurons innervating extensor digitorum brevis were preferentially affected than those innervating abductor halluces brevis, in accordance with previous studies (14, 15). In the present study, the frequency of A-waves and frequency of subjects with A-waves in the ulnar and tibial nerves were significantly increased in the ALS patients, in accordance with the split-hand and split-leg syndrome in ALS (16, 17). In addition, the significantly slower disease progression rate of the ALS patients with A-waves than that of the ALS patients without A-waves supported that the occurrence of A-waves may imply a slower rate of the degenerative process and a relatively better prognosis. No correlation was found between characteristics of A-wave and F-wave in ALS in the present study, possibly the amplitude and persistence of F-waves were affected by dysfunction of both upper and lower motoneurons in ALS (18). The pathological severity between nerves with early A-waves and nerves without early A-waves was comparable, which probably indicated that the A-wave latencies had no correlations with the disease severity.

There were some limitations of the present study. In the present study, disease duration was defined as time between symptom onset and date of evaluation. However, the time of symptom onset dictated by patients might be inaccurate. Better way is to follow up those patients at least once by experienced physicians, and then evaluate the disease progression rate. The study employed a series of 100 F-waves in each nerve, which was difficult to perform in usual clinical practice. Further study is needed to explore the characteristics of A-waves by a moderate number of stimuli. What’s more, the exact determination of the frequency of A-waves was rather difficult. There may be an underestimation of the actual frequency due to the following reasons. (1) The frequency of more than 35% responses out of 100 stimuli necessary to accept a response to be an A-wave was arbitrary. Therefore, those investigations with fewer than 35% recordings showing identical responses that occurred in some of our studies were not taken into account. (2) Since the present study employed a supramaximal stimulation to elicit F-waves, A-waves that were axon reflexes may be suppressed. (3) In some cases, A-waves with very short latencies may not be detected using superficial recording as they were a part of the M-response. (4) It was sometimes difficult to determine A-waves that were part of the F-wave complex. (5) In patients with ALS, the number of repeater F-waves was significantly increased (18), and there were difficulties in differentiating repeater F-waves from A-waves, especially when A-waves occurred among the F-waves.

In conclusion, the present study demonstrated that A-waves could be detected in patients with ALS. The presence of A-waves would imply a dysfunction of lower motor neurons and possibly support a relatively slower disease progression rate, hence a better prognosis.

## Ethics Statement

The research protocol was approved by the ethics committee of Peking Union Medical College Hospital and adhered to the principles of Declaration of Helsinki.

## Author Contributions

JF and LC conceived and designed the experiments; JF and LC performed the experiments; JF, LC, ML, YG, QD, and DS analyzed the data; ML, YG, QD, HT, and DL contributed reagents/materials/analysis tools; JF and LC contributed to the writing of the manuscript.

## Conflict of Interest Statement

The authors declare that the research was conducted in the absence of any commercial or financial relationships that could be construed as a potential conflict of interest.
